# Energy-dense dietary patterns high in free sugars and saturated fat and associations with obesity in young adults

**DOI:** 10.1007/s00394-021-02758-y

**Published:** 2021-12-06

**Authors:** Katherine Mary Livingstone, Meaghan J. Sexton-Dhamu, Felicity J. Pendergast, Anthony Worsley, Barbara Brayner, Sarah A. McNaughton

**Affiliations:** 1 Institute for Physical Activity and Nutrition (IPAN), School of Exercise and Nutrition Sciences, Deakin University, Geelong, Australia; 2 Department of Medicine, Cedars-Sinai Medical Center, Los Angeles, CA USA; 3 School of Exercise and Nutrition Sciences, Deakin University, Geelong, Australia; 4 Institute for Physical Activity and Nutrition (IPAN), School of Exercise and Nutrition Sciences, Deakin University, Melbourne Burwood Campus, 221 Burwood Highway, Burwood, VIC 3125 Australia

**Keywords:** Obesity, Dietary patterns, Energy density, Fiber density, Saturated fat, Adults

## Abstract

**Purpose:**

To derive dietary patterns based on dietary energy density (DED), free sugars, SFA, and fiber and investigate association with odds of overweight/obesity in young adults.

**Methods:**

Cross-sectional data from 625 young Australian adults (18–30 years) were used. Dietary patterns were derived using reduced rank regression based on dietary data from a smartphone food diary using DED, free sugars, SFA, and fiber density as response variables. Multivariable logistic regression was used to investigate associations between dietary patterns and odds of self-reported overweight/obesity (BMI ≥ 25 kg/m^2^).

**Results:**

Two dietary patterns were identified (DP1 and DP2). DP-1 was positively correlated with DED, free sugars, and SFA, and inversely correlated with fiber density. It was characterized by higher sugar-sweetened beverages intake and lower vegetable intake, and associated with higher odds of overweight/obesity (OR: 1.22; 95% CI 1.05, 1.42). DP-2 was positively correlated with fiber density and free sugars, and inversely correlated with DED and SFA. It was characterized by higher sugar-sweetened beverages intake and lower non-lean red meat intake, and was not significantly associated with overweight/obesity.

**Conclusion:**

An energy-dense dietary pattern high in free sugars and SFA and low in fiber was associated with higher odds of obesity in young adults. These findings support dietary interventions that target reductions in energy-dense foods and sugar-sweetened beverages.

**Supplementary Information:**

The online version contains supplementary material available at 10.1007/s00394-021-02758-y.

## Introduction

Diets high in energy-dense foods, such as processed meats, as well as high energy sugar-sweetened beverages have been associated with a greater risk of obesity and chronic disease [1]. Young adults often have energy-dense diets due to social norms, a preference for packaged and convenience foods, and low cooking confidence [2–4]. Moreover, young adults are the most at-risk age group for weight gain and developing chronic diseases in later life; by 2030, it is predicted that 80% of young Australian adults will have overweight or obesity [5]. Identification of risk factors for obesity in young adulthood will inform the design of dietary interventions to reduce the tracking of unhealthy diets and chronic disease risk into later life.

Dietary pattern methodologies, such as reduced rank regression, consider the frequency and combination of foods consumed, providing a more holistic approach to characterizing overall diets rather than focusing on single foods and nutrients [6]. Reduced rank regression is a hybrid approach that enables the creation of food-based dietary patterns that explain variation in multiple nutrient intakes known to be associated with a specific disease [7, 8], providing a policy-relevant link between nutrient and food-based guidelines [9]. With increasing consensus that energy-dense diets high in free sugars and saturated fats and low in fiber are associated with obesity risk [10], there is a need to examine these within the context of an overall dietary pattern.

Few studies have derived dietary patterns based on energy density, fat, and/or fiber intake and investigated their associations with obesity risk [11, 12]; and those that have, have focused on adolescence and demonstrated mixed results. In a cross-sectional study of 1251 Australian adolescents aged 17 years, an energy-dense dietary pattern high in total fat and low in fiber was associated with higher waist circumference, but not BMI, in girls only. [11] In contrast, a cross-sectional analysis of European and Australian adolescent samples identified a similar dietary pattern associated with BMI in boys. [13] Since autonomous eating behaviors are established in young adulthood, [14] understanding the specific foods that characterize an energy-dense diet in this age group, as well as associations with overall diet quality and obesity, will inform targeted dietary interventions and policies to improve diets and health outcomes. To our knowledge, no studies have incorporated free sugars into the creation of such dietary patterns. Free sugars include all sugars added by the manufacturer, cook, or consumer and sugars naturally present in juiced or pureed fruit and vegetables, but exclude sugars that naturally occur in dairy products and intact fruit and vegetables [15]. Free sugars, as opposed to total or added sugars, have been identified as most strongly associated with poor health outcomes, including obesity, and are a key component of World Health Organization dietary recommendations [10, 15]. With the high intake of free sugars in young adults, understanding their role as part of an overall dietary patterns will provide new insights into young adult eating behaviors. Thus, this study aimed to derive dietary patterns based on dietary energy density (DED), percentage of energy (%E) from free sugars and SFA, and fiber density in young Australian adults and to investigate their cross-sectional associations with sociodemographic characteristics, diet quality and odds of overweight/obesity.

## Methods

### Subjects and study design

This study used data from the Measuring Eating in everyday Life Study (MEALS), a cross-sectional study conducted between April 2015 and April 2016 to examine eating patterns in young adults [3, 16]. Participants were aged between 18 and 30 years, living in Victoria, Australia. Pregnant or lactating women were considered ineligible to participate in the study because of potential changes in their eating patterns [17]. Recruitment was conducted using social media (Facebook, Twitter), poster advertisements, and flyers. After providing written consent, participants completed an online questionnaire (via Qualtrics, an online questionnaire hosting platform) and the FoodNow real-time, food diary smartphone application (app). Participants were compensated for their time with a $25 voucher. Reporting was guided by the STROBE-nut guidelines for nutritional epidemiology (Table S1).

## Study measures

### Dietary intake

Participants used the FoodNow app to record all foods and beverages they consumed over four non-consecutive days (3 weekdays and 1 weekend day). To represent usual intake and to maximize our sample, participants were excluded from the present analysis if they did not complete three or four days of food diaries. Data were collected over an eight-day period to account for daily variations in food consumption. Before each eating occasion, participants were encouraged to take two images of their food and beverage items alongside a standardized business card [18]. If it was not possible to take an image, participants could bypass this step by selecting ‘No image’. Participants were then prompted to provide a text description containing information such as the type, brand, and amount of food they consumed, and had the option to provide a voice recording. The text description was mandatory, whereas the collection of images and voice recordings was optional but highly encouraged by research staff. Following each eating occasion, participants were prompted by the FoodNow app to answer a food wastage question and take an image of any leftover food items to control for over-reporting of food consumption. Participants were provided with a detailed instruction manual, frequently asked questions and a YouTube video tutorial demonstrating how to download and use the FoodNow app. These instructions also provided guidance on how to report the amount of a food or drink consumed, with a variety of possible measurement techniques. Trained nutritionists or dietitians coded the food intake data against the Food Standards Australia New Zealand AUSNUT 2011–13 food composition database and a purpose designed database was used to calculate nutrient intake from the FoodNow app [19]. The standardized business card was used as a size reference (fiducial marker) when assessing the images to assist with coding the amount of food or drink consumed. Total energy intake (kJ/day), %E from total fat, SFA, monounsaturated fat (MUFA), polyunsaturated fat (PUFA), protein, carbohydrates, total sugars and free sugars, and intake of dietary fiber (g/day), sodium (mg/day), calcium (mg/day), iron (mg/day), and vitamin C (mg/day) were estimated. These nutrients were selected to reflect the main macro- and micro-nutrients correlated with food groups used to create the dietary patterns (e.g., calcium to reflect dairy product intake and vitamin C to reflect fruit and vegetable intake). In an evaluation of the FoodNow app in a sample of 90 young adults, energy intake measured by the FoodNow app has demonstrated good agreement against objectively measured energy expenditure [18]. DED (kJ/100 g) was calculated by dividing total food energy (kJ) by total food weight (g), excluding all beverages, and multiplying by 100. The decision to exclude beverages was based on evidence that measuring DED using both food and beverages may disproportionately affect DED values and lessen the strength of associations with outcome variables [20]. Average food and nutrient intakes were estimated for each participant using the mean of the three or four days of intake from the FoodNow app.

### Dietary patterns

Information on food and beverage intakes from the FoodNow app was used to estimate dietary patterns. Fifty food and beverage groups (g/day) were created based on comparable nutrient compositions and the AUSNUT 2011–13 food group classification system [19] (Table S2). Foods were categorized into the following food groups: fruit (*n* = 5), vegetables (*n* = 4), cereals (*n* = 8), fats and oils (*n* = 2), dairy (*n* = 7), meats and alternatives *(n* = 10), soups, sauces, spreads, and condiments (*n* = 4), snacks and confectionery (*n* = 5), non-alcoholic beverages (*n* = 2), and alcoholic beverages (*n* = 3).

Dietary patterns were derived using reduced rank regression. Reduced rank regression derives linear combinations (factors) of food and beverage intake data that maximize the explained variation in intermediate response variables that are hypothesized to be related to a particular health outcome [7]. The number of dietary patterns extracted depends on the number of response variables and consistent with previous application of reduced rank regression, only dietary patterns that represented more than 10% of the variation in the response variables were used in regression analyses [21]. In these analyses, four nutrients known to be associated with obesity risk were used as response variables: DED (kJ/100 g), fiber density (g/MJ), free sugars (%E), and SFA (%E). These nutrients were selected based on evidence from the World Health Organization’s report on chronic disease prevention that DED, fiber density, free sugars, and fat intakes are strongly associated with obesity risk [10, 22]. Previous research has provided evidence to support using these variables when assessing obesity risk [12, 23].

Sensitivity analyses were performed to test the robustness of the derived dietary patterns. First, usual dietary intake was derived based on three or four days of FoodNow data using the web-based statistical program Multiple Source Method [24]. Usual dietary intake was estimated by (1) including consumption frequency as a covariate using population-specific proportions of food group intake from the Australian Health Survey [25] and (2) assuming all individuals were habitual consumers. Second, new dietary patterns were created in a random 50% split sample. Tucker’s coefficient of congruence was used to assess comparability between the dietary patterns [26] derived in the full sample using average intakes, usual intakes, and a 50% split sample. A coefficient of *r* ≥ 0.95 was considered equal, while *r* in the range of 0.85 to 0.94 indicated fair similarity. The coefficient of congruence was also calculated to assess the effect of excluding energy intake misreporters on the reproducibility of the dietary patterns.

### Diet quality

To determine whether dietary patterns were associated with overall diet quality, diet quality was estimated from food and beverage intake data from the FoodNow app using the Dietary Guideline Index (DGI). The DGI is a food-based diet quality index designed to reflect consumption according to the 2013 Australian Dietary Guidelines (Table S3) and is detailed elsewhere [27]. Briefly, the DGI-2013 comprises ten recommended dietary components (food variety, fruit, vegetables, total and wholegrain cereals, total and lean meat and alternatives, dairy and alternatives, and total beverage and water intake) and six discouraged dietary components (discretionary foods, SFA, added salt, extra sugar, and alcohol) [27]. As added salt was not recorded in the FoodNow app, this component of the DGI-2013 was omitted for this study. DGI-2013 component scores ranged between 0 and 10, and sub-component scores ranged between 0 and 5. Cut-offs for the components were guided by the Australian Dietary Guidelines’ age- and sex-specific recommendations. [27] Total DGI scores ranged between 0 and 120, with higher scores representing better diet quality.

### Weight status

Participants were asked to self-report their weight (kg) and height (cm) in the online questionnaire [16]. Body mass index (BMI) was calculated as weight (kg)/height (m^2^). Overweight/obesity was classified as a binary variable using standard cut-offs of underweight/healthy weight (BMI < 25 kg/m^2^) and overweight/obesity (BMI ≥ 25 kg/m^2^). [28]

### Confounders

Confounders were determined based on the existing literature and a DAGitty directed acyclic graph (Figure S1). Information on country of birth, education status, postcode, physical activity, and smoking habits were collected using the online questionnaire. Country of birth was operationalized as born in Australia or other. Education status was grouped into Year 12 or less, trade/certificate, and tertiary [29]. The Socio-Economic Indexes for Areas (SEIFA) Index for Relative Socio-economic Disadvantage was used to assess the socioeconomic status of participants [30]. Participants’ postcodes were used to assign a SEIFA score derived using low socioeconomic attribute data from the 2018 Australian Census such as low income and education and unskilled occupations. The SEIFA score was operationalized as low (most disadvantaged), medium, and high (least disadvantaged). Physical activity was collected using the International Physical Activity Questionnaire—Short Form. [31] Participants’ level of physical activity was assessed according to whether they met the Australian physical activity guidelines of at least 150 min of moderate-intensity or 75 min of vigorous-intensity physical activity per week [32]. Smoking habits were grouped as non-smoking or past/current smoker, as research suggests that the dietary behaviors of past and current smokers are similar [33]. Energy misreporting was determined by dividing energy intake (from the FoodNow app) by predicted total energy expenditure, applying age- and sex-specific equations to a BMI range, and assuming a ‘low active’ physical activity level of  ≥ 1.4 and < 1.6. [34] For sensitivity analyses purposes, participants who were classified as misreporters (i.e., under-reporters and over-reporters) were identified based on the ± 1 SD (standard deviation) cut-off for energy misreporting [34].

### Statistical analyses

Complete case analysis was used. Means and SD (continuous data) or percentages (categorical data) were used for descriptive statistics. Dietary patterns were treated as continuous variables in regression analyses. Dietary patterns were also categorized into tertiles for descriptive purposes and to confirm that regression analyses were consistent when treated as a continuous or categorical variable. For construct validity, linear regression was used to examine associations between dietary patterns (continuous; independent variables) and DGI, total energy and nutrient intakes (continuous; dependent variables) and were adjusted for age and sex. Multivariable linear regression was used to examine associations between dietary patterns (as continuous and tertiles; independent variables) and BMI (continuous; dependent variable). Multivariable logistic regression was used to examine associations between the dietary patterns (as continuous and tertiles; independent variables) and overweight/obesity (binary, dependent variable). These analyses were adjusted for age (continuous), sex (binary), country of birth (binary), level of education (categorical), SEIFA (categorical), physical activity (binary), smoking (binary), and energy misreporting (continuous). As sensitivity analyses, these regression analyses were run after excluding energy misreporters to determine the effect, if any, on the strength and direction of associations between dietary patterns, overweight/obesity and BMI. SAS (version 9.4; SAS Institute, Cary, NC) was used to derive the dietary patterns. Stata (version 16; StataCorp., College Station, TX, USA) was used to analyze the data. Statistical significance was set at *p* < 0.05.

## Results

Of the 983 participants recruited into MEALS, 675 participants were included in the present analyses (mean age 24.3 [3.5 SD] years, 72% female). Most participants were born in Australia (72%), highly educated (69%), had low area-level disadvantage (61%), and met physical activity recommendations (64%). Twenty-four percent of participants had overweight or obesity. Participants were excluded if they did not complete the online questionnaire (*n* = 89), did not complete three or four days of dietary data (*n* = 85), or had missing data in the questionnaire (*n* = 17; Figure S2). The proportion of participants who completed 3 and 4 days of FoodNow data were 13 and 87%, respectively. Participant characteristics (age, sex) were comparable between three and four days (data not shown). Images and voice recordings were provided by 72 and 16% of the participants, respectively, with 16% providing both. Thirty percent of participants were identified as misreporting their energy intake (*n* = 103 under-reporting; *n* = 100 over-reporting). The excluded and included samples were similar concerning participants’ age, country of birth, SEIFA, and smoking status; however, more young adults in the included sample were female or tertiary educated (Table S4).

## Dietary patterns

Table 1 presents the explained variation in food intakes and response variables and the correlations between dietary patterns, referred to as DP, and response variables. DP-1 was moderately positively correlated with DED (*r* = 0.54) and %E intake from SFA (*r* = 0.45) and moderately inversely correlated with fiber density (*r* = –0.60). It was also weakly positively correlated with %E intake from free sugars (*r* = 0.38). DP-2 was strongly positively correlated with %E intake from free sugars (*r* = 0.90). It was also weakly positively correlated with fiber density (*r* = 0.17) and weakly inversely correlated with %E intake from SFA (*r* = –0.37) and DED (*r* = –0.14). DP-3 and DP-4 were not further investigated, as they explained < 10% of the variation in response variables.

**Table 1:** Explained variation (%) in food intakes and nutrient intake response variables for each dietary pattern and correlation coefficients between dietary patterns and nutrient intake response variables (*n* = 675)^a^

	Explained variation (%)						Correlation coefficient			
	Total		Nutrient response variables							
	Food intake	Nutrient response variables	DED (kJ/100 g)	Fiber density (g/MJ)	Free sugars (%E)	SFA (%E)	DED (kJ/100 g)	Fiber density (g/MJ)	Free sugars (%E)	SFA (%E)
DP-1	4.64	36.1	42.7	51.7	21.1	28.8	0.54*	− 0.60*	0.38*	0.45*
DP-2	2.43	12.9	43.7	53.1	63.0	36.0	− 0.14	0.17	0.90*	− 0.37*
DP-3	3.13	6.9	48.5	56.6	64.2	54.1	− 0.42*	0.36*	0.20	0.81*
DP-4	2.48	4.1	56.8	64.5	64.2	54.2	0.71*	0.70*	0.01	0.06

*DED* dietary energy density, *DP* dietary pattern

*Correlation coefficient is significant at *P* < 0.001

DP-1 was characterized by higher intakes of sugar-sweetened beverages, cakes and sweet biscuits and savory products and lower intakes of green and brassica vegetables, pome, berry, and stone fruit and other vegetables. DP-2 was characterized by higher intakes of sugar-sweetened beverages, fruit and vegetable juices and high-sugar products (e.g., chocolate spread) and lower intakes of animal-based fats, non-lean red meat and eggs (Table 2; Tables S4 and S5).

**Table 2:** Intakes of response variables and top five positive and inverse loading food groups across tertiles of dietary patterns (*n* = 675)^a^

Food groups	Factor loading	Tertile of dietary pattern			*P *trend^b^
		Tertile 1	Tertile 2	Tertile 3	
Dietary pattern 1					
Response variables					
DED (kJ/100 g)	–	592 ± 111	701 ± 137	875 ± 183	< 0.001
Fiber density (g/MJ)	–	4.34 ± 1.08	3.23 ± 0.69	2.46 ± 0.86	< 0.001
Free sugars (%E)	–	4.92 ± 2.85	6.48 ± 4.12	9.64 ± 5.41	< 0.001
SFA (%E)	–	9.99 ± 2.72	11.6 ± 2.64	13.8 ± 3.35	< 0.001
Positive associations (g/day)					< 0.001
Sugar-sweetened beverages	0.30	29.5 ± 68.2	46.5 ± 79.1	170 ± 239	< 0.001
Cakes and sweet biscuits	0.24	10.31 ± 20.5	17.3 ± 26.9	31.0 ± 39.0	< 0.001
Savory products	0.23	117 ± 112	165 ± 135	213 ± 155	< 0.001
Chocolate	0.19	3.21 ± 6.95	3.88 ± 8.52	9.13 ± 16.2	< 0.001
Butter and animal-based fats	0.18	1.05 ± 2.62	1.06 ± 2.34	2.87 ± 5.83	< 0.001
Inverse associations					
Green/brassica vegetables	− 0.29	123 ± 104	59.4 ± 64.5	46.2 ± 47.0	< 0.001
Pome/berry/stone fruit	− 0.28	89.9 ± 76.0	47.8 ± 48.0	26.9 ± 49.5	< 0.001
Other (whole) vegetables	− 0.23	93.6 ± 81.8	53.7 ± 55.7	39.7 ± 50.3	< 0.001
Legumes/beans	− 0.22	38.3 ± 62.5	16.0 ± 35.7	7.58 ± 20.8	< 0.001
Wholegrains	− 0.20	50.6 ± 49.5	28.0 ± 32.5	23.4 ± 30.6	< 0.001
Dietary pattern 2					
Response variables					
DED (kJ/100 g)	–	765 ± 179	699 ± 189	705 ± 187	0.001
Fiber density (g/MJ)	–	3.03 ± 1.01	3.57 ± 1.22	3.44 ± 1.23	< 0.001
Free sugars (%E)	–	4.32 ± 3.02	6.19 ± 3.27	10.5 ± 5.08	< 0.001
SFA (%E)	–	13.0 ± 3.20	11.5 ± 3.37	11.0 ± 3.03	< 0.001
Positive associations (g/day)					
Sugar-sweetened beverages	0.48	24.9 ± 64.8	40.0 ± 89.1	181 ± 229.3	< 0.001
Fruit/vegetable juices	0.38	7.45 ± 25.4	21.2 ± 51.3	68.1 ± 103.3	< 0.001
High-sugar products	0.31	2.31 ± 3.34	3.27 ± 5.45	8.02 ± 11.7	< 0.001
Dairy desserts	0.21	9.44 ± 20.3	12.6 ± 24.0	23.8 ± 48.4	< 0.001
Cakes and sweet biscuits	0.18	13.8 ± 25.0	16.0 ± 26.0	28.9 ± 38.1	< 0.001
Inverse associations (g/day)					
Butter and animal-based fats	− 0.25	2.79 ± 5.93	1.15 ± 2.52	1.04 ± 2.27	< 0.001
Non-lean, red meat	− 0.21	25.1 ± 34.9	13.1 ± 23.4	11.8 ± 20.9	< 0.001
Eggs	− 0.18	33.2 ± 49.3	18.7 ± 26.8	15.0 ± 27.7	< 0.001
High-fat cheeses	− 0.17	17.2 ± 19.5	12.3 ± 17.2	10.5 ± 16.5	< 0.001
Lean poultry	− 0.15	75.6 ± 84.1	50.0 ± 67.7	46.6 ± 68.6	< 0.001

*DED* dietary energy density

^a^Values represent mean ± SD

^b^Linear regression analyses examined associations across tertiles of dietary pattern. Analyses were adjusted for age and sex

As shown in Table S7, DP-1 was associated with higher total energy intake and intake of total fat, SFA, free sugars, and sodium and lower intake of PUFA, dietary fiber, iron, and vitamin C. DP-2 was associated with higher intake of carbohydrates, total sugars, free sugars, dietary fiber, and vitamin C and lower intake of total fat, SFA, MUFA, PUFA, and protein. DP-1, but not DP-2, was associated with lower DGI.

Sensitivity analyses using Tucker’s coefficient of congruence indicated that factor loadings for dietary patterns derived when using average intakes were comparable to the factor loadings for average and usual intakes using a full and 50% split sample (*r* = 0.94–1.00). The coefficient of congruence also indicated similarities between the factor loadings for dietary patterns using average intakes and the sample of valid energy intake reporters (i.e., excluding energy misreporters; *r* = 0.91–0.94). Tables S5 and S6 provide the factor loadings for the dietary patterns derived using usual intakes, 50% split sample and in valid energy intake reporters.

## Dietary patterns and demographics characteristics

Demographic characteristics of individuals across DP tertiles are provided in Table 3. Participants with a higher DP-1 score were more likely to be male, live in areas with higher area-level disadvantage, and not meet physical activity recommendations. Individuals with a higher DP-2 score were younger, female, and born in Australia.

**Table 3:** Demographic characteristics across tertiles of dietary patterns (*n* = 675)^a^

Characteristic	All	Dietary pattern			*P* trend^b^
		Tertile 1	Tertile 2	Tertile 3	
Dietary pattern 1					
Dietary pattern score	0.00 ± 0.29	− 1.33 ± 0.81	0.00 ± 0.28	1.33 ± 0.83	< 0.001
Age (years)	24.3 ± 3.53	24.6 ± 3.48	24.0 ± 3.45	24.2 ± 3.64	0.25
Female (%)	72.3	79.6	66.7	70.7	0.008
Country of birth (%)					
Australia	75.3	75.1	72.0	78.7	0.26
Other	24.7	24.9	28.0	21.3	
Highest level of education (%)					
Low	28.3	25.3	29.8	29.8	0.36
Medium	13.2	11.1	12.9	15.6	
High	58.5	63.6	57.3	54.7	
SEIFA (%)					
Low	13.6	11.1	10.7	19.1	0.021
Medium	25.5	23.1	25.8	27.6	
High	60.9	65.8	63.6	53.3	
Smoking (%)					
Never/former smoked	80.6	80.0	80.4	81.3	0.94
Current smoker	19.4	20.0	19.6	18.7	
Physically active (%)	63.7	73.3	58.2	59.6	0.001
BMI (kg/m^2^)	23.2 ± 4.37	22.5 ± 3.39	23.3 ± 4.42	23.8 ± 5.07	0.001
BMI category (%)					
Underweight/normal weight	76.4	80.4	77.3	71.6	0.079
Overweight/obesity	23.6	19.6	22.7	28.4	
Dietary pattern 2					
Dietary pattern score	0.00 ± 1.00	− 0.99 ± 0.55	– 0.08 ± 0.20	1.07 ± 0.72	< 0.001
Age, years	24.3 ± 3.53	24.7 ± 3.53	24.2 ± 3.50	23.8 ± 3.49	0.004
Female, %	72.3	64.0	78.7	74.2	0.002
Country of birth					
Australia	75.3	67.6	74.2	84.0	< 0.001
Other	24.7	32.4	25.8	16.0	
Highest level of education					
Low	28.3	25.8	27.6	31.6	0.48
Medium	13.2	11.6	13.8	14.2	
High	58.5	62.7	58.7	54.2	
SEIFA					
Low	13.6	14.7	12.4	13.8	0.35
Medium	25.5	26.7	21.3	28.4	
High	60.9	58.7	66.2	57.8	
Smoking (%)					
Never/former smoked	80.6	80.0	77.3	84.4	0.16
Current smoker	19.4	20.0	22.7	15.6	
Physically active (%)	63.7	64.0	64.9	62.2	0.84
BMI (kg/m^2^)	23.2 ± 4.37	23.3 ± 3.64	22.9 ± 3.89	23.4 ± 5.39	0.79
BMI category (%)					
Underweight/normal weight	76.4	77.3	77.3	74.7	0.74
Overweight/obesity	23.6	22.7	22.7	25.3	

*BMI* body mass index, *SEIFA* Socio-economic index for areas

^a^Values represent mean ± SD. Education: low (completed some high-school or less), medium (completed high-school or completed some high-school and/or certificate/diploma) and high (having a tertiary qualification). BMI category: underweight/ normal weight (BMI < 25 kg/m^2^), overweight (25 ≤ BMI < 30 kg/m^2^), obesity (BMI ≥ 30 kg/m^2^); SEIFA: low (most disadvantaged), medium and high (least disadvantaged). Physically active was defined according to whether they met the Australian physical activity recommendations of at least 150 min of moderate-intensity or 75 min of vigorous-intensity physical activity per week

^b^Linear regression analyses (continuous variables) and χ^2^ (categorical variables) were used to examine trends across tertiles

## Dietary patterns, obesity, and BMI

DP-1 was positively associated with odds of overweight/obesity (OR: 1.22; 95% CI 1.05, 1.42) and positively associated with BMI. A one-unit increase in DP-1 was associated with a 0.55 kg/m^2^ (SD 0.13) increase in BMI. Results were comparable when DP-1 was treated as a categorical variable (Table 4). DP-2 was not significantly associated with odds of overweight/obesity or BMI.

**Table 4:** Association between dietary patterns and overweight/obesity and body mass index (*n* = 675)

	Overweight/obesity^a^			Body mass index^b^		
	Odds ratio	95% CI	*P *value	Beta coeff	SD	*P *trend
Dietary pattern 1						
Continuous	1.22	1.05, 1.42	0.01	0.55	0.13	< 0.001
Tertile 1 (ref)	1.00	–		1.00	–	
Tertile 2	1.12	0.70, 1.80	0.64	0.59	0.40	0.14
Tertile 3	1.58	1.00, 2.50	0.05	1.23	0.40	0.002
Dietary pattern 2						
Continuous	1.13	0.94, 1.36	0.21	0.12	0.17	0.46
Tertile 1 (ref)	1.00	–		1.00	–	
Tertile 2	1.05	0.66, 1.68	0.83	–0.30	0.41	0.46
Tertile 3	1.27	0.80, 2.00	0.31	0.27	0.41	0.50

^a^Logistic regression was used to examine associations between the dietary pattern 1 and 2 (as continuous and tertiles) and overweight/obesity (binary). Analyses were adjusted for age (not when used to stratify), sex, country of birth, education, Socio-economic Index for areas, smoking, physical activity, and energy misreporting

^b^Linear regression was used to examine associations between dietary pattern 1 and 2 (as continuous and tertiles) and body mass index (continuous). Analyses were adjusted for age (not when used to stratify), sex, country of birth, education, Socio-economic Index for areas, smoking, physical activity, and energy misreporting

As sensitivity analyses to examine the robustness of these associations, we investigated whether excluding energy misreporters from the analyses influenced the relationship between dietary patterns and overweight/obesity and BMI. Associations were similar after excluding participants who had misreported their energy intake (Table S8). DP-1 remained positively associated with odds of overweight/obesity (OR: 1.23; 95% CI 1.02, 1.48) and positively associated with BMI, with a one-unit increase in DP-1 being associated with a 0.56 kg/m^2^ (SD 0.25) increase in BMI. The non-significant association remained between DP-2 and odds of overweight/obesity and BMI.

## Discussion

This study used a real-time food diary smartphone app to identify an energy-dense dietary pattern high in free sugars and SFA and low in fiber that was associated with higher odds of overweight/obesity in young adults. The young adults who consumed this dietary pattern had a lower overall diet quality and were more likely to be male and live in areas of greater disadvantage, highlighting the need to better support these young adults to make healthier choices. Moreover, the design of dietary interventions in young adults would benefit from specifically targeting reductions in energy-dense foods high in free sugars and SFA and sugar-sweetened beverages.

The energy-dense dietary pattern identified in this study is consistent with the high intake of energy-dense foods and beverages among adolescents and young adults reported in other studies [35–37]. Data from the 2011–2013 Australian Health Survey indicate that the average DED of diets in Australian young adults aged 18–34 years is 740 kJ/100 g (vs. 723 kJ/100 g in our study), showing the generalizability of this pattern with the wider young adult cohort in Australia [35]. Moreover, the foods that characterized the dietary pattern in the present study were comparable with the high DED foods identified in these national data, including high intake of sweet biscuits and chocolate. The highest loading item in the dietary pattern identified in our study was sugar-sweetened beverages, which is known to be consumed most by young adult age groups [37]. Some energy-dense foods are also nutrient-dense (e.g., cheese and nuts), and the absence of these foods loading highly in this pattern suggests it is nutrient-poor dietary pattern. This is further supported by the inverse correlations with healthful nutrients, such as iron and vitamin C, which are likely attributable to low intake of lean meat and fruit and vegetables, respectively. Moreover, the inverse association with this dietary pattern and overall diet quality is consistent with our previous findings linking diet quality and obesity [38].

Research examining associations between energy-dense diets and obesity risk has shown mixed results [11, 39]. Direct comparisons with previous studies are challenging due to the lack of studies in young adults that have derived dietary patterns using reduced rank regression. A study in a nationally representative sample of US adults aged 20 years and over showed strong evidence that DED is positively associated with BMI and waist circumference [39], while two studies in adolescents that derived similar energy-dense and high-fat dietary patterns using reduced rank regression showed mixed associations with BMI. The authors of these studies postulated that mixed results may be partly due to the choice of response variables. As most research to date has focused on DED, SFA and fiber density, the present study is the first to derive dietary patterns based on free sugars intake. As free sugars intake has been implicated with greater risk of weight gain and type 2 diabetes than intake of total or added sugars [15], these findings will help inform future dietary interventions to improve diets in young adults. With high amounts of free sugars consumed globally [15], further research should determine the reproducibility of these dietary patterns in other young adult cohorts and examine their prospective associations with obesity.

Sugar-sweetened beverages were the highest positive loading item in both dietary patterns identified in this study. Previous research in MEALS has shown that sugar-sweetened beverages were consumed most often in the home and often while undertaking screen-based activities, such as watching TV [40], suggesting these may be appropriate targets for behavioral intervention to reduce intake in young adults. Only DP-1 was associated with lower diet quality and higher odds of overweight/obesity in this study, while these associations were not observed for DP-2. This difference in findings between DP-1 and DP-2 is likely attributable to the weak loadings for fruit and vegetables and wholegrain cereals in DP-2 which are high in fiber and can contribute to diets of lower energy density. In contrast, these foods were strongly inversely loaded in DP-1, as well as loading highly for SFA-rich foods, suggesting that very low intakes of fruit and vegetables and wholegrain cereals, coupled with high intakes of high-SFA and sugars foods and beverages. may be an integral component of the dietary pattern, consistent with lower diet quality, and higher odds of obesity. Regardless, these findings support a focus of interventions targeting reductions in sugar-sweetened beverages for reducing obesity risk, but also add to the wider body of literature on the adverse health outcomes associated with diets high in sugar-sweetened beverages, such as cardiovascular and oral health [41, 42].

These findings have the potential to inform food-based dietary guidelines for obesity prevention in young adults as they show the importance of considering foods and beverages as part of an overall dietary pattern. Our results reinforce recommendations to increase consumption of nutrient-dense foods high in fiber, such as vegetables, while minimizing energy-dense foods high in free sugars and SFA, such as cakes and sweet biscuits. Given the comparable strength of the factor loadings for these energy-dense foods (positively loaded) and nutrient-dense foods (inversely loaded) in the pattern, there is significant potential for developing dietary interventions that focus on swapping strategies, such as snacking on vegetables instead of cakes and sweet biscuits. In particular, a recent systematic review of dietary strategies has highlighted the benefits of individuals swapping energy-dense foods for nutrient-dense foods [43], supporting a focus on shifting this energy-dense dietary pattern toward a nutrient-dense pattern to improve overall diets and reduce obesity prevalence.

The present study has several strengths. A validated real-time food diary smartphone app was used to collect information on dietary intake. While all self-report dietary assessment methods involve measurement error [44], the FoodNow app was shown to have good agreement with objective measures of energy expenditure. Moreover, providing images as part of a dietary assessment tool was appropriate for use in this age group, as indicated by the majority of participants providing images [45]. This is consistent with research demonstrating that images can improve the accuracy of dietary assessment tools [46]. A further strength is the use of reduced rank regression to derive dietary patterns. By combining a priori and a posteriori information, this method can derive patterns of food group intake that may be more appropriate for the diseases of interest. Moreover, by examining multiple response variables, this technique can capture multiple dietary intake dimensions that reflect the underlying complexity of diet. However, this may also increase the complexity of interpretations and although sensitivity analyses demonstrated the robustness of the patterns identified, these patterns are specific to the dietary intake of the cohort and are limited in their generalizability to other cohorts. Future research should examine the reproducibility of these dietary patterns by replicating them in an independent young adult cohort.

Several limitations should be acknowledged. First, our findings are not generalizable to the wider young adult population, as the sample was predominantly female, tertiary educated, and from areas of low area-level disadvantage. Although a lack of diversity is common in research from self-selected samples in this age range [47, 48], further efforts are required to recruit diverse samples. Second, the self-report nature of the dietary and anthropometric data may be subject to misreporting biases, and future research should consider collecting data on waist circumference. Third, reverse causality cannot be discounted due to the cross-sectional study design, and although we adjusted for key confounders known to influence our associations, residual or unmeasured confounding may remain, such as sedentary behavior. Prospective research is needed to examine whether the energy-dense dietary patterns identified in this study are associated with the development of obesity in older adulthood.

This study identified an energy-dense dietary pattern high in free sugars and SFA and low in fiber that was associated with higher odds of obesity in young adults. Findings from this novel smartphone-based study offer insights into real-time methods for examining dietary patterns in young adults. These findings also inform the design of policies and interventions to improve diets in this critical age group; if intervened in young adulthood, improved diets have the potential to track into later adulthood and to improve their future health and that of their children. To improve overall dietary patterns in young adults, future research should design dietary interventions that target reductions in these energy-dense foods and sugar-sweetened beverages.

## Supplementary Information

Below is the link to the electronic supplementary material.

Supplementary file1 (DOCX 180 KB)

Supplementary file2 (DOCX 33 KB)

Supplementary file3 (DOCX 24 KB)

Supplementary file4 (DOCX 49 KB)

## Data Availability

The datasets generated and/or analyzed during the current study are not publicly available due to Deakin University’s Human Research Ethics Committee procedures but are available from the senior author on reasonable request.

## Abbreviations

BMI: Body mass index
DGI: Dietary guideline index
DP: Dietary pattern
DED: Dietary energy density
MEALS: Measuring eating in everyday life study
MUFA: Monounsaturated fat
PUFA: Polyunsaturated fat
SFA: Saturated fat
SEIFA: Socio-economic index for areas

## Code availability

NA.
