# Supplementary material for: Energy-dense dietary patterns high in free sugars and saturated fat and associations with obesity in young adults
Source: Eur J Nutr. 2021 Dec 6;61(3):1595–607. doi: 10.1007/s00394-021-02758-y (PMC8921009; doi:10.1007/s00394-021-02758-y)
Supplement: Supplementary file 1 — Supplementary file1 (DOCX 180 KB) [file 394_2021_2758_MOESM1_ESM.docx]

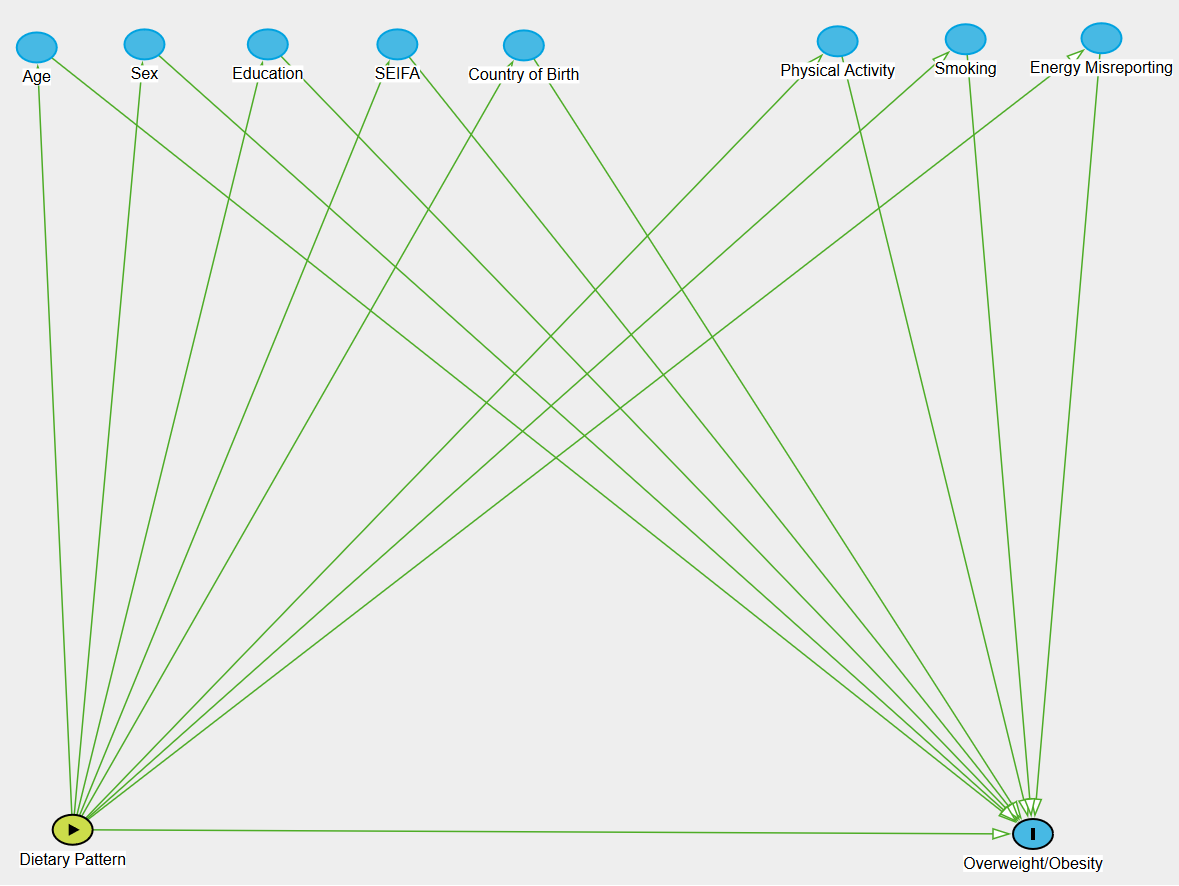


Figure S1

Directed acyclic graph of the association between dietary patterns and overweight/obesity

Energy-dense dietary patterns high in free sugars and saturated fat and associations with obesity in young adults

Katherine M. Livingstone, Institute for Physical Activity and Nutrition, School of Exercise and Nutrition Sciences, Deakin University, Geelong, Australia [k.livingstone@deakin.edu.au](mailto:k.livingstone@deakin.edu.au)
