# Supplementary material for: Energy-dense dietary patterns high in free sugars and saturated fat and associations with obesity in young adults
Source: Eur J Nutr. 2021 Dec 6;61(3):1595–607. doi: 10.1007/s00394-021-02758-y (PMC8921009; doi:10.1007/s00394-021-02758-y)
Supplement: Supplementary file 2 — Supplementary file2 (DOCX 33 KB) [file 394_2021_2758_MOESM2_ESM.docx]

Completed 3 or 4 days of FoodNow diaries (n=692)

Excluded (n=85)

- Did not meet inclusion criteria of 3 or 4 days of dietary data

Consenting participants in the MEALS (n=983)

Excluded (n=141)

- Did not complete online questionnaire (n=101)
- Withdrew/ineligible after completing questionnaires (n=40)

Completed online questionnaires (n=842)

Excluded due to missing data* (n=17)

- Sex (n=11)
- SEIFA (n=4)
- BMI (n=2)

Used FoodNow diaries (n=777)

Participants included in analysis (n=675)

**Figure S2**

STROBE-nut participant flow diagram.

*, Variables not mutually exclusive

Energy-dense dietary patterns high in free sugars and saturated fat and associations with obesity in young adults

Katherine M. Livingstone, Institute for Physical Activity and Nutrition, School of Exercise and Nutrition Sciences, Deakin University, Geelong, Australia [k.livingstone@deakin.edu.au](mailto:k.livingstone@deakin.edu.au)
