# Supplementary material for: Energy-dense dietary patterns high in free sugars and saturated fat and associations with obesity in young adults
Source: Eur J Nutr. 2021 Dec 6;61(3):1595–607. doi: 10.1007/s00394-021-02758-y (PMC8921009; doi:10.1007/s00394-021-02758-y)
Supplement: Supplementary file 4 — Supplementary file4 (DOCX 49 KB) [file 394_2021_2758_MOESM4_ESM.docx]

**Table S2** Food groups used as predictors in the reduced rank regression analyses (n=50)

| **Food Categories** | **Food Groups** |
| --- | --- |
| Fruit (five groups) | Pome, berry and stone fruit |
|  | Citrus fruit |
|  | Topical and other fruit |
|  | Dried fruit |
|  | Fruit or vegetable juice |
| Vegetables (four groups) | Green and brassica vegetables |
|  | Orange vegetables |
|  | Starchy vegetables (e.g., potato, sweet potato and sweetcorn) |
|  | Other (whole) vegetables |
| Cereals | Wholegrain/high-fibre breads, grains and flours |
|  | Wholegrain/high-fibre oats and breakfast cereals |
|  | Wholegrain/high-fibre savoury crackers, crumpets, English muffins, scones |
|  | Refined/low-fibre breads, grains and flours |
|  | Refined/low-fibre oats and breakfast cereals |
|  | Refined/low-fibre savoury crackers, crumpets, English muffins, scones |
|  | Cakes and sweet biscuits |
|  | Savoury pastries, biscuits or products (e.g., pies, pizza and hot chips) |
| Fats and oils (two groups) | Unsaturated margarine and oils |
|  | Butter, animal-based solid fats and dairy blends |
| Dairy (seven groups) | High-fat/moderate-fat cheese, hard, soft and fresh |
|  | Moderate-fat milk, milk alternatives and calcium products |
|  | Moderate-fat yoghurt and dairy-based yoghurts |
|  | Low-fat milk, milk alternatives and calcium products |
|  | Low-fat cheese, hard, soft and fresh |
|  | Low-fat yoghurt and dairy-based yoghurts |
|  | Cream and condensed milk |
| Meats and alternatives (10 groups) | Red meat, lean |
|  | Red meat, non-lean (including processed lean red meats) |
|  | Poultry, lean |
|  | Poultry, non-lean (including processed lean poultry) |
|  | Fish and seafood |
|  | Battered fish and fish products |
|  | Eggs |
|  | Legumes/beans |
|  | Tofu |
|  | Nuts and seeds |
| Soups, sauces, spreads and condiments (four groups) | Soups, homemade, dry mix and canned |
|  | Sauces |
|  | Spreads, dips and high-fat dressings |
|  | Condiments |
| Snacks and confectionary (five groups) | Snack foods (e.g., potato snacks and corn snacks) |
|  | Jam, honey and high-sugar products |
|  | Chocolate |
|  | Cereal and protein bars |
|  | Dairy desserts and other confectionary |
| Non-alcoholic beverages (two groups) | Tea, coffee and water |
|  | Fruits drinks, sports drinks, cordials, soft drinks, sugar-sweetened beverages |
| Alcoholic beverages (three groups) | Beers and ciders |
|  | Wines |
|  | Spirits and other alcoholic beverages |

**Table S3** Dietary Guideline Index (DGI) 2013 components and scoring methods

| **DGI component** | **Scoring criteria** | |
| --- | --- | --- |
|  | **Minimum score criteria (0)** | **Maximum score criteria (10)*** |
| **Encouraged components** | | |
| 1. Enjoy a wide variety of nutritious foods |  |  |
| Food variety: variety of foods consumed within the core food groups |  |  |
| 2. Plenty of vegetables |  |  |
| Total vegetable intake: servings of vegetables per day | M: 0, F: 0 | M: ≥6, F: ≥5 |
| 3. Fruit |  |  |
| Total fruit intake: servings of fruit per day | 0 | ≥2 |
| 4. Grain foods |  |  |
| (a). Total cereal intake: servings of grains per day | 0 | ≥6 |
| (b). Mostly wholegrain or high-fibre cereals (proportion of wholegrain/high-fibre to refined/low-fibre cereals or grains) | 0% | 100% |
| 5. Lean meat and poultry, fish, eggs, nuts and seeds, and legumes/beans |  |  |
| (a). Total meat and alternative: servings per day | M: 0, F: 0 | M: ≥3, F: ≥2.5 |
| (b). Lean meat: proportion of lean meats and alternatives to total meat and alternatives per day | 0% | 100% |
| 6. Milk, yoghurt, cheese and/or their alternatives (serves per day) |  |  |
| Total dairy and alternative: servings per day | M: 0, F: 0 | M: ≥3, F: ≥2.5 |
| 7. Drink plenty of water |  |  |
| (a). Total beverage intake: servings per day | M: 0, F: 0 | M: ≥10, F: ≥8 |
| (b). Water: proportion of water to total beverage intake per day | 0% water | ≥50% water |
| **Discouraged components** | | |
| 8. Limit intake of foods containing saturated fat, added salt, added sugars and alcohol |  |  |
| Limit discretionary foods | M: >3, F: >2.5 | M: ≤3, F: ≤2.5 |
| 9. Limit intake of foods high in saturated fat. |  |  |
| (a). Trimmed meat: trimming fat from meat | Never or rarely | Usually |
| (b). Choose reduced-fat milk: type of milk usually consumed | Whole milk | Skim/low/reduced-fat milk |
| 10. Small allowance of unsaturated oils, fats or spreads |  |  |
| Unsaturated spreads and oils: servings per day | M: >4, F: >2 | M: ≤4, F: ≤2 |
| 11. Limit intake of foods and drinks containing added sugars |  |  |
| 12. If you choose to drink alcohol, limit intake |  |  |
| Limit alcohol: servings per day | >2 | ≤2 |
| **Total DGI-2013 Score** | 0 | 120 |

Note. * Sub-components are scored a maximum of 5, components are scored a maximum of 10. M=male, F=female.

**Table S4** Demographic characteristics of excluded and included participants (n=842)^1^

| Characteristic | Excluded sample (n=167) | Included sample (n=675) | P-value^2^ |
| --- | --- | --- | --- |
| Age, y | 24.0 ± 3.6 | 24.3 ± 3.5 | 0.41 |
| Female, % | 52.8 | 72.3 | <0.0001 |
| Country of birth, % |  |  |  |
| Australia | 76.7 | 75.3 | 0.71 |
| Other | 23.3 | 24.7 |  |
| Highest level of education, % |  |  |  |
| Low | 34.1 | 28.3 | 0.12 |
| Medium | 16.2 | 13.2 |  |
| High | 49.7 | 58.5 |  |
| SEIFA, % |  |  |  |
| Low | 16.0 | 13.6 | 0.74 |
| Medium | 25.2 | 25.5 |  |
| High | 58.9 | 60.9 |  |
| Smoking, % |  |  |  |
| Never/former smoked | 74.9 | 80.6 | 0.10 |
| Current smoker | 25.2 | 19.4 |  |

1, Values represent mean ± SD.

2, Linear regression analyses (continuous variables) and χ^2^ (categorical variables) were used to test for associations between samples.

Excluded sample smaller for sex (n=78) and SEIFA (n=4).

**Table S5** Dietary pattern 1 factor loadings of food groups for the full sample (n=675) using average intake and MSM usual intakes as well as for a random split sample (n=337)

| Food groups^1^ | Factor loading | | |
| --- | --- | --- | --- |
|  | Full sample average | Full sample MSM | Split sample^2^ |
| Positive loading |  |  |  |
| Sugar-sweetened beverages | **0.30** | **0.28** | **0.30** |
| Cakes and sweet biscuits | **0.24** | **0.26** | **0.21** |
| Savoury products | **0.23** | **0.24** | **0.27** |
| Chocolate | **0.19** | **0.19** | **0.15** |
| Butter, animal-based fats and blends | **0.18** | **0.19** | **0.16** |
| Dairy desserts and other confectionery | **0.18** | **0.21** | **0.19** |
| Non-lean red meat | **0.15** | **0.18** | 0.14 |
| Non-lean poultry | 0.14 | 0.14 | **0.17** |
| Beers and ciders | 0.13 | 0.13 | **0.17** |
| High-fat/moderate-fat cheeses | 0.12 | 0.13 | 0.10 |
| Moderate-fat milk and alternatives | 0.12 | 0.11 | 0.11 |
| Cream and condensed milk | 0.11 | 0.11 | 0.03 |
| Snack foods (e.g., potato snacks and corn snacks) | 0.11 | 0.12 | 0.08 |
| Spirits and other alcoholic beverages | 0.08 | 0.07 | 0.14 |
| Jam, honey and high-sugar products | 0.06 | 0.06 | 0.03 |
| Fruit and vegetable juice | 0.06 | 0.05 | 0.04 |
| Lean poultry | 0.05 | 0.06 | 0.06 |
| Refined oats and breakfast cereals | 0.05 | 0.05 | 0.02 |
| Refined breads, grains and flours | 0.04 | 0.06 | 0.04 |
| Refined savoury products | 0.03 | 0.04 | 0.01 |
| Lean red meat | 0.03 | 0.05 | 0.05 |
| Sauces | 0.03 | 0.04 | –0.01 |
| Cereal and protein bars | 0.01 | 0.02 | 0.04 |
| Wines | 0.01 | 0.02 | 0.05 |
| Unsaturated margarine and oils | 0.00 | 0.00 | –0.06 |
| Negative loading |  |  |  |
| Green/brassica vegetables | **–0.29** | **–0.28** | **–0.32** |
| Pome, berry and stone fruit | **–0.28** | **–0.28** | **–0.25** |
| Other (whole) vegetables | **–0.23** | **–0.22** | **–0.21** |
| Legumes and beans | **–0.22** | **–0.22** | **–0.19** |
| Wholegrain/high-fibre breads, grains and flours | **–0.20** | **–0.17** | **–0.19** |
| Orange vegetables | **–0.18** | **–0.18** | **–0.18** |
| Starchy vegetables | **–0.18** | **–0.18** | **–0.22** |
| Wholegrain/high-fibre oats and breakfast cereals | **–0.16** | –0.14 | –0.14 |
| Tropical and other fruit | **–0.15** | **–0.15** | –0.12 |
| Low-fat milk and milk alternatives | **–0.15** | **–0.15** | **–0.22** |
| Nuts and seeds | –0.14 | **–0.15** | **–0.15** |
| Tofu | –0.14 | –0.13 | –0.14 |
| Citrus fruit | –0.13 | –0.14 | –0.12 |
| Soups (homemade, dry mix and canned) | –0.13 | –0.11 | –0.14 |
| Tea, coffee and water | –0.11 | –0.09 | –0.12 |
| Fish and seafood | –0.10 | –0.10 | –0.05 |
| Dried fruit | –0.09 | –0.10 | –0.08 |
| Low-fat yoghurt and dairy-based yoghurt | –0.09 | –0.08 | –0.03 |
| Wholegrain/high-fibre savoury products | –0.05 | –0.05 | –0.07 |
| Spreads, dips and high-fat dressings | –0.05 | –0.03 | –0.03 |
| Low-fat cheeses | –0.04 | –0.03 | –0.06 |
| Eggs | –0.01 | –0.01 | –0.04 |
| Condiments | –0.01 | 0.01 | –0.04 |
| Battered fish and fish products | –0.00 | 0.03 | –0.02 |
| Moderate-fat yoghurt | –0.00 | 0.01 | –0.03 |

1, Reduced rank regression was used to derive dietary patterns. Food groups represent the top five highest loading food groups with direct and inverse associations. Factor loadings greater than 0.15 are bolded.

2, 50% split sample of average dietary intakes

**Table S6** Dietary pattern 2 factor loadings of food groups for the full sample (n=675) using average intake and MSM usual intakes as well as for a random split sample (n=337)

| Food groups^1^ | Factor loading | | |
| --- | --- | --- | --- |
|  | Full sample average | Full sample MSM | Split sample^2^ |
| Positive loading |  |  |  |
| Sugar-sweetened beverages | **0.48** | **0.42** | **0.49** |
| Fruit and vegetable juices | **0.38** | **0.39** | **0.36** |
| Jam, honey and high-sugar products | **0.31** | **0.34** | **0.30** |
| Dairy desserts and other confectionary | **0.21** | **0.27** | **0.20** |
| Cakes and sweet biscuits | **0.18** | **0.26** | **0.25** |
| Low-fat milk and milk alternatives | **0.15** | **0.19** | 0.07 |
| Legumes and beans | 0.14 | 0.13 | **0.22** |
| Chocolate | 0.14 | **0.20** | 0.14 |
| Tropical and other fruit | 0.14 | **0.17** | 0.06 |
| Spirits and other alcoholic beverages | 0.13 | 0.10 | **0.18** |
| Pome, berry and stone fruit | 0.13 | **0.16** | 0.14 |
| Wholegrain/high-fibre oats and breakfast cereals | 0.11 | 0.14 | **0.16** |
| Moderate-fat milk and milk alternatives | 0.11 | **0.16** | 0.14 |
| Refined oats and breakfast cereals | 0.10 | 0.08 | –0.04 |
| Refined savoury products | 0.08 | 0.10 | 0.02 |
| Tofu | 0.06 | 0.06 | 0.06 |
| Sauces | 0.06 | 0.09 | 0.01 |
| Snack foods (e.g., potato snacks and corn snacks) | 0.04 | 0.06 | 0.01 |
| Low-fat yoghurt and dairy-based yoghurt | 0.04 | 0.05 | 0.10 |
| Moderate-fat yoghurt and dairy-based yoghurt | 0.04 | 0.08 | –0.00 |
| Cereal and protein bars | 0.03 | 0.08 | –0.02 |
| Citrus fruit | 0.03 | 0.04 | 0.03 |
| Dried fruit | 0.03 | 0.05 | 0.07 |
| Spreads, dips and high-fat dressings | 0.03 | 0.08 | 0.08 |
| Orange vegetables | 0.01 | 0.05 | –0.00 |
| Starchy vegetables | 0.00 | 0.02 | 0.01 |
| Negative loading |  |  |  |
| Butter, animal-based fats and dairy blends | **–0.25** | **–0.15** | **–0.15** |
| Non-lean, red meat | **–0.21** | **–0.16** | **–0.21** |
| Eggs | **–0.18** | **–0.16** | **–0.18** |
| High-fat/moderate-fat cheeses | **–0.17** | –0.07 | **–0.15** |
| Lean poultry | **–0.15** | **–0.15** | **–0.17** |
| Nuts and seeds | –0.13 | –0.09 | –0.10 |
| Fish and seafood | –0.10 | –0.07 | –0.12 |
| Refined breads, grains and flours | –0.10 | –0.11 | –0.14 |
| Cream and condensed milk | –0.10 | –0.04 | –0.11 |
| Beers and ciders | –0.09 | –0.06 | –0.06 |
| Lean, red meat | –0.08 | –0.05 | –0.03 |
| Non-lean poultry | –0.08 | –0.08 | –0.02 |
| Unsaturated margarine and oils | –0.08 | –0.02 | –0.07 |
| Wines | –0.05 | –0.03 | –0.06 |
| Savoury products (e.g., pastries, pies and pizza) | –0.04 | –0.04 | –0.07 |
| Other (whole) vegetables | –0.04 | 0.01 | –0.06 |
| Wholegrain/high-fibre savoury products | –0.03 | –0.02 | 0.01 |
| Battered fish and fish products | –0.03 | –0.04 | –0.04 |
| Green and brassica vegetables | –0.03 | 0.01 | –0.01 |
| Condiments | –0.03 | 0.01 | –0.01 |
| Wholegrain/high-fibre breads, grains and flours | –0.03 | –0.00 | 0.05 |
| Tea, coffee and water | –0.02 | 0.05 | –0.01 |
| Low-fat cheeses (hard, soft and fresh) | –0.01 | 0.02 | –0.04 |
| Soups (handmade, dry mix and canned) | –0.01 | 0.05 | 0.00 |

1, Reduced rank regression was used to derive dietary patterns. Food groups represent the top five highest loading food groups with direct and inverse associations. Factor loadings greater than 0.15 are bolded.

2, 50% split sample of average dietary intake

**Table S7** Diet quality and nutrient intakes across tertiles of dietary patterns (n=675)^1^

|  | All | Dietary pattern 1 | | | | Dietary pattern 2 | | | | |
| --- | --- | --- | --- | --- | --- | --- | --- | --- | --- | --- |
|  |  | Tertile 1 | Tertile 2 | Tertile 3 | P value^2^ | | Tertile 1 | Tertile 2 | Tertile 3 | P value^2^ |
| Dietary Guideline Index | 62.4 ± 13.5 | 71.9 ± 10.6 | 61.9 ± 11.1 | 53.5 ± 11.8 | <0.0001 | | 61.5 ± 12.7 | 63.9 ± 13.4 | 61.9 ± 14.2 | 0.61 |
| Total energy (kJ) | 7449 ± 3010 | 7235 ± 2292 | 7003 ± 3068 | 8109 ± 3450 | 0.009 | | 7920 ± 3298 | 6586 ± 2485 | 7841 ± 3013 | 0.50 |
| Macronutrients |  |  |  |  |  | |  |  |  |  |
| Total fat (%E) | 34.6 ± 6.5 | 33.4 ± 6.6 | 34.2 ± 6.2 | 36.1 ± 6.3 | <0.0001 | | 37.5 ± 6.9 | 34.1 ± 5.8 | 32.2 ± 5.5 | <0.0001 |
| Saturated fat (%E) | 11.8 ± 3.3 | 10.0 ± 2.7 | 11.6 ± 2.6 | 13.8 ± 3.4 | <0.0001 | | 13.0 ± 3.2 | 11.5 ± 3.4 | 11.0 ± 3.0 | <0.0001 |
| Mono-unsaturated fat (%E) | 13.7 ± 3.4 | 13.6 ± 3.6 | 13.5 ± 3.5 | 13.8 ± 3.1 | 0.51 | | 14.9 ± 3.9 | 13.6 ± 3.2 | 12.5 ± 2.7 | <0.0001 |
| Poly-unsaturated fat (%E) | 6.1 ± 2.1 | 6.7 ± 2.5 | 6.1 ± 1.8 | 5.5 ± 1.6 | <0.0001 | | 6.4 ± 2.5 | 6.1 ± 1.9 | 5.9 ± 1.8 | 0.008 |
| Protein (%E) | 18.9 ± 4.5 | 19.7 ± 5.1 | 19.1 ± 4.2 | 18.0 ± 3.9 | <0.0001 | | 20.3 ± 4.6 | 19.0 ± 4.4 | 17.5 ± 3.9 | <0.0001 |
| Total carbohydrates (%E) | 42.9 ± 7.9 | 42.7 ± 8.4 | 43.2 ± 7.9 | 42.9 ± 7.3 | 0.94 | | 38.6 ± 7.6 | 43.4 ± 6.9 | 46.8 ± 6.8 | <0.0001 |
| Total sugars (%E) | 16.4 ± 6.2 | 16.7 ± 6.0 | 15.6 ± 5.9 | 17.0 ± 6.5 | 0.45 | | 12.0 ± 4.5 | 16.8 ± 4.9 | 20.5 ± 5.8 | <0.0001 |
| Free sugars (%E) | 7.0 ± 4.7 | 4.9 ± 2.9 | 6.5 ± 4.1 | 9.6 ± 5.4 | <0.0001 | | 4.3 ± 3.0 | 6.2 ± 3.3 | 10.5 ± 5.1 | <0.0001 |
| Dietary fibre (g) | 23.6 ± 10.9 | 30.2 ± 10.7 | 21.6 ± 9.3 | 19.1 ± 9.3 | <0.0001 | | 22.7 ± 10.7 | 22.6 ± 10.8 | 25.6 ± 10.9 | <0.0001 |
| Micronutrients |  |  |  |  |  | |  |  |  |  |
| Sodium (mg) | 2251 ± 1109 | 2052 ± 855 | 2204 ± 1184 | 2498 ± 1212 | <0.0001 | | 2465 ± 1261 | 2002 ± 935 | 2286 ± 1063 | 0.37 |
| Calcium (mg) | 731 ± 374 | 752 ± 333 | 679 ± 390 | 763 ± 392 | 0.99 | | 741 ± 389 | 676 ± 340 | 778 ± 385 | 0.09 |
| Iron (mg) | 10.4 ± 4.8 | 11.9 ± 4.5 | 9.7 ± 4.6 | 9.7 ± 5.1 | <0.0001 | | 10.7 ± 5.1 | 9.6 ± 4.6 | 10.9 ± 4.6 | 0.14 |
| Vitamin C (mg) | 96.6 ± 67.8 | 134 ± 77.3 | 85.0 ± 56.8 | 70.9 ± 49.3 | <0.0001 | | 82.7 ± 55.3 | 93.8 ± 66.5 | 113 ± 76.7 | <0.0001 |

1, Values represent mean ± SD. Diet quality was assessed using the 2013 Dietary Guideline Index

2, Linear regression analyses were used to test for trends across tertiles and were adjusted for age and sex

**Table S8** Association between dietary patterns and overweight/obesity and body mass index, excluding misreporters (n=472)

|  | Overweight/obesity^1^ | | | Body mass index^2^ | | |
| --- | --- | --- | --- | --- | --- | --- |
|  | Odds ratio | 95% CI | *P-*value | Beta coeff | SD | *P-*trend |
| Dietary pattern 1 |  |  |  |  |  |  |
| Continuous | 1.23 | 1.02, 1.48 | 0.031 | 0.56 | 0.15 | <0.001 |
| Tertile 1 (ref) | 1.00 | - | - | 1.00 | - | - |
| Tertile 2 | 1.24 | 0.70, 2.19 | 0.46 | 0.99 | 0.47 | 0.037 |
| Tertile 3 | 1.69 | 0.97, 2.92 | 0.06 | 1.59 | 0.47 | 0.001 |
| Dietary pattern 2 |  |  |  |  |  |  |
| Continuous | 1.18 | 0.94, 1.49 | 0.16 | 0.17 | 0.20 | 0.41 |
| Tertile 1 (ref) | 1.00 | - | - | 1.00 | - | - |
| Tertile 2 | 1.40 | 0.80, 2.46 | 0.24 | 0.20 | 0.48 | 0.68 |
| Tertile 3 | 1.38 | 0.79, 2.41 | 0.26 | 0.53 | 0.48 | 0.26 |

1, Logistic regression was used to examine associations between the dietary pattern 1 and 2 (as continuous and tertiles) and overweight/obesity (binary). Analyses were adjusted for age (not when used to stratify), sex, country of birth, education, Socio-economic Index for Areas, smoking, physical activity, and energy misreporting.

2, Linear regression was used to examine associations between dietary pattern 1 and 2 (as continuous and tertiles) and body mass index (continuous). Analyses were adjusted for age (not when used to stratify), sex, country of birth, education, Socio-economic Index for Areas, smoking, physical activity, and energy misreporting.

Energy-dense dietary patterns high in free sugars and saturated fat and associations with obesity in young adults

Katherine M. Livingstone, Institute for Physical Activity and Nutrition, School of Exercise and Nutrition Sciences, Deakin University, Geelong, Australia [k.livingstone@deakin.edu.au](mailto:k.livingstone@deakin.edu.au)
